# L'atteinte vésicale au cours de la neurofibromatose de Von Recklinghausen

**DOI:** 10.11604/pamj.2014.17.294.4152

**Published:** 2014-04-18

**Authors:** Mohamed Hicham Benazzouz, Tilila Hajjad, Younes Essatara, Hachem El Sayegh, Ali Iken, Lounis Benslimane, Yassine Nouini

**Affiliations:** 1 Service d'Urologie A, Hôpital Ibn Sina, CHU Rabat, Maroc; 2 Service de Chirurgie Plastique et Réparatrice, Hôpital Ibn Sina, CHU Rabat, Maroc

**Keywords:** Neurofibromatose, maladie de Von Recklinghausen, vessie, Neurofibromatosis, Von Recklinghausen disease, bladder

## Abstract

La neurofibromatose de type 1 ou maladie de Von Recklinghausen est une maladie génétique autosomique dominante en rapport avec des mutations dans le gène suppresseur de tumeur NF1. L'atteinte uro-génitale au cours de cette maladie est rare et moins de 80 cas ont été rapportés à ce jour dans la littérature mondiale. Les auteurs rapportent un nouveau cas d'atteinte vésicale découverte fortuitement au cours du suivi d'une patiente atteinte de la maladie de Von Recklinghausen. A travers cette observation et une revue de la littérature les auteurs discutent également les difficultés diagnostiques, thérapeutiques ainsi que les modalités de suivi dans cette maladie.

## Introduction

La neurofibromatose de type 1 ou maladie de Von Recklinghausen est une maladie génétique autosomique dominante, en rapport avec des mutations dans le gène suppresseur de tumeur NF1, prédisposant les sujets atteints à une variété de tumeurs bénignes (neurofibromes et neurofibromes plexiformes) et malignes [1, 2]. L'atteinte uro-génitale au cours de la maladie de Von Recklinghausen est rare et moins de 80 cas ont été rapporté à ce jour dans la littérature mondiale [3]. Cette atteinte peut intéresser le pénis, le clitoris, la prostate, l'urètre, les testicules, les cordons spermatiques et les uretères. Cependant la vessie reste l'organe le plus atteint [3, 4]. Les auteurs rapportent un nouveau cas d'atteinte vésicale découverte fortuitement chez une patiente suivie pour maladie de Von Recklinghausen. A travers cette observation et une revue de la littérature les auteurs se proposent de discuter les complications ainsi que les difficultés thérapeutiques et de suivi dans cette neurofibromatose.

## Patient et observation

**Figure 1:**
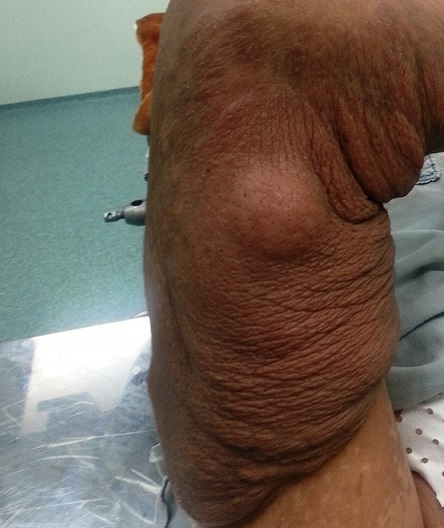
Tumeur royale au niveau de la face postérieure du bras gauche

M.A, patiente âgée de 22 ans, sans antécédents pathologiques particuliers, s'est présentée au service de chirurgie plastique pour prise en charge d'une tumeur au niveau du membre supérieur gauche. L'examen retrouvait une tumeur royale prenant la face postérieure du bras et l'avant bras gauche, de consistance molle et de couleur plus foncée que la peau normale (Figure 1). Le reste de l'examen somatique retrouvait plus d'une vingtaine de taches café au lait disséminées dans tout le corps sans aucun autre signe associé. Une exérèse chirurgicale de la tumeur du membre supérieure gauche a été réalisée. L'examen anatomo-pathologique était en faveur d'un neurofibrome diffus et plexiforme de l'avant bras. Devant la présence de plus de six taches café au lait et d'un neurofibrome plexiforme, le diagnostic de neurofibromatose de Von Recklinghaussen a été retenu chez cette patiente.

**Figure 2:**
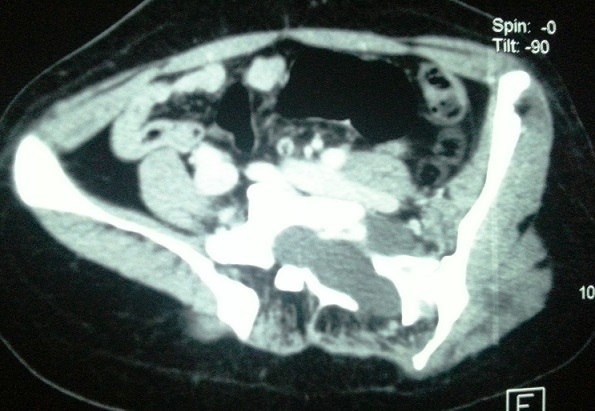
Coupe scannographique montrant une lésion kystique lombaire intracanalaire avec extension extra-foraminale évoquant un neurofibrome

**Figure 3:**
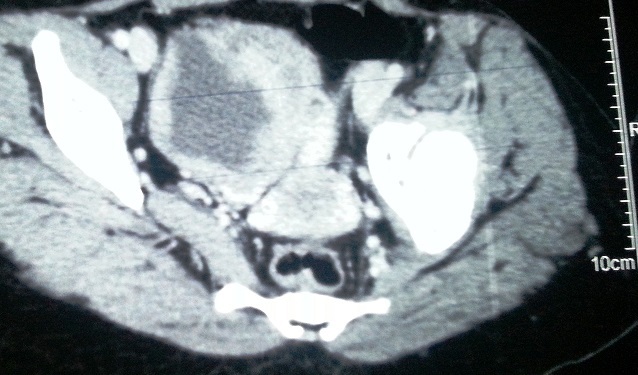
Coupe scannographique montrant un épaississement de la paroi vésicale postéro-latérale bilatérale se rehaussant après injection de produit de contraste

Au cours de son suivi cette patiente a présenté un nodule sous cutané au niveau de la fesse gauche. Une TDM abdomino-pelvienne a été réalisé dans le but d'apprécier la nature du nodule fessier et afin d’évaluer ses rapports avec les structures de voisinage. Cette TDM a permis de découvrir de manière fortuite des lésions kystiques intra-canalaires, étendues sur tout le rachis lombaire, évoquant des neurofibromes (Figure 2) ainsi qu'un épaississement de la paroi vésicale postéro-latérale bilatérale mesurant 16mm (Figure 3), rehaussé après injection de produit de contraste et sans retentissement sur le haut appareil urinaire. Une cystoscopie réalisée retrouvait une vessie de bonne capacité, des méats urétéraux en situation normale avec une muqueuse vésicale parfaitement saine sans masse bourgeonnante en endoluminale. Devant l'absence de symptomatologie urinaire, de retentissement sur le haut appareil urinaire et de signes radiologiques suspects de malignité une surveillance rapprochée a été décidée chez cette patiente. Cette surveillance associe une échographie vésicale chaque 3 mois réalisée par le même opérateur, l'alternance de TDM et d'IRM tout les 6 mois dans un but de radioprotection ainsi que la réalisation d'une cystoscopie une fois par an ou en cas de modification radiologique ou devant l'apparition d'une symptomatologie urinaire.

## Discussion

La neurofibromatose de type 1 ou maladie de Von Recklinghausen est une maladie génétique très fréquente qui touche 1/4000 à 1/3000 individus [1, 4]. C'est une affection autosomique dominante en rapport avec des mutations dans le gène suppresseur de tumeur NF1 prédisposant les sujets atteints à une variété de tumeurs bénignes (neurofibromes et neurofibromes plexiformes) et malignes [1, 2].

C'est une maladie dont l'expression clinique est très variable mais dont le diagnostic est essentiellement clinique avec la présence de 2 ou plus des critères suivants (critères NIH - Bethesda, 1988) [5]: Un apparenté du premier degré atteint (parent, fratrie ou enfant) - Au moins 6 taches café au lait (TCLs) > 1,5 cm après la puberté ou > 0,5 cm avant la puberté - Lentigines axillaires ou inguinales -Ou:.Au moins deux neurofibromes quel que soit le type. Au moins un neurofibrome plexiforme - Gliome du nerf optique - Au moins 2 nodules de Lisch (hamartome irien) - Une lésion osseuse caractéristique: Pseudarthrose, dysplasie du sphénoïde, amincissement du cortex des os longs.

La grande variabilité clinique ainsi que le risque de survenue de tumeurs bénignes et malignes imposent un suivi régulier chez ces patients, que ce soit, clinique ou radiologique. Ce qui est le cas de notre patiente.

L'atteinte uro-génitale au cours de la maladie de Von Recklinghausen est rare. Elle peut intéresser le pénis, le clitoris, la prostate, l'urètre, les testicules, les cordons spermatiques et les uretères. Cependant la vessie reste l'organe le plus atteint [3, 4].

Sur le plan clinique, l'atteinte vésicale peut se traduire par une symptomatologie irritative, une dysurie voir une rétention d'urines et parfois une hématurie[3, 4]. D'autres signes cliniques tels que les lombalgies en rapport avec un retentissement sur le haut appareil urinaire ont également été rapportés [3, 4]. Ces signes cliniques traduisent la présence de neurofibromes qui sont des tumeurs bénignes qui se développent le long des gaines nerveuses mais aussi dans les plexi péri-vasculaires de la vessie. Le risque de transformation maligne de ces neurofibromes en neurofibrosarcomes est faible mais à redouter [4]. Ce risque est estimé à 12-29% et augmente avec l’âge [3, 4]. D'autres tumeurs malignes peuvent également toucher la vessie. Il s'agit des: paragangliomes, léiomyosarcomes, carcinomes transitionnel et des rhabdomyosarcomes [4].

Du point de vue radiologique, l’échographie retrouve le plus souvent un épaississement pariétal diffus ou localisé. La TDM et l'IRM sont souvent nécessaires pour mieux caractériser ces anomalies et pour évaluer l'extension tumorale. Sur la TDM les neurofibromes sont homogènes et hypodenses par rapport aux muscles adjacents [6]. Alors que sur l'IRM les neurofibromes sont homogènes, iso-signal T1 par rapport aux muscles, hyper signal T2 entourés par une bande hyposignal [6].

L'IRM est très utile pour la détection précoce d'une éventuelle transformation maligne. En faveur de la malignité, on retient l'asymétrie des lésions, l'hétérogénéité tumorale et l'intensité de la prise de contraste [7].

Le traitement de l'atteinte vésicale au cours de la maladie de Von Recklinghausen n'est pas bien codifié. L'indication thérapeutique dépend de la symptomatologie clinique, l'existence d'un éventuel retentissement sur le haut appareil urinaire et la suspicion d'une dégénérescence maligne.

Chez les patients asymptomatiques, la surveillance doit être privilégiée [3]. Le rythme de cette surveillance reste à déterminer. Cependant une surveillance rapprochée par des cystoscopies et des examens d'imagerie (TDM et IRM) semble être l'attitude la plus raisonnable étant donné le risque de dégénérescence maligne.

Les patients présentant des signes irritatifs du bas appareil urinaire devraient bénéficier d'un traitement anti-cholinergique tant que possible [3]. En cas de progression de la maladie avec l'apparition de signes obstructifs du bas appareil urinaire le recours à la chirurgie devient nécessaire [2, 3]. Un traitement conservateur par une résection trans-urétrale ou une cystectomie partielle semble être le plus approprié. En cas d'obstruction du haut appareil urinaire, une dérivation urinaire devient impérative. L'examen histologique extemporané de l'extrémité distal des uretères est nécessaire pour ne pas laisser de la tumeur résiduelle [3]. L'exérèse chirurgicale de la masse doit être préférée dans ces cas là car elle permettrait un diagnostic histologique plus précis.

Enfin, devant une tumeur maligne découverte sur l'examen histologique des copeaux de résection trans-urétrale ou d'une pièce de cystectomie partielle, ou devant des signes radiologiques fortement suspect de malignité, une cystectomie radicale devra être envisagée.

## Conclusion

L'atteinte vésicale au cours de la maladie de Von Recklinghausen est rare. Dans les formes asymptomatiques et en l'absence de signes radiologiques suspects de malignité une surveillance semble être le traitement de choix. Les formes symptomatiques quant à elle relève d'un traitement chirurgical qui doit être aussi conservateur que possible. Dans tout les cas une surveillance rapprochée est nécessaire compte tenu du risque de dégénérescence maligne qui n'est pas négligeable. Les moyens et le rythme de cette surveillance reste à préciser.
